# Land Cover Classification of UAV Remote Sensing Based on Transformer–CNN Hybrid Architecture

**DOI:** 10.3390/s23115288

**Published:** 2023-06-02

**Authors:** Tingyu Lu, Luhe Wan, Shaoqun Qi, Meixiang Gao

**Affiliations:** 1 College of Geographical Sciences, Harbin Normal University, Harbin 150025, China; lutingyu@sina.com (T.L.); qsq-00@163.com (S.Q.); 2 Heilongjiang Province Key Laboratory of Geographical Environment Monitoring and Spatial Information Service in Cold Regions, Harbin Normal University, Harbin 150025, China; 3 Department of Geography and Spatial Information Techniques, Ningbo University, Ningbo 315211, China; gmx102@163.com

**Keywords:** deep learning, transformer, CNN, semantic segmentation, UAV remote sensing

## Abstract

High-precision land cover maps of remote sensing images based on an intelligent extraction method are an important research field for many scholars. In recent years, deep learning represented by convolutional neural networks has been introduced into the field of land cover remote sensing mapping. In view of the problem that a convolution operation is good at extracting local features but has limitations in modeling long-distance dependence relationships, a semantic segmentation network, DE-UNet, with a dual encoder is proposed in this paper. The Swin Transformer and convolutional neural network are used to design the hybrid architecture. The Swin Transformer pays attention to multi-scale global features and learns local features through the convolutional neural network. Integrated features take into account both global and local context information. In the experiment, remote sensing images from UAVs were used to test three deep learning models including DE-UNet. DE-UNet achieved the highest classification accuracy, and the average overall accuracy was 0.28% and 4.81% higher than UNet and UNet++, respectively. It shows that the introduction of a Transformer enhances the model fitting ability.

## 1. Introduction

In recent years, remote sensing of unmanned aerial vehicles (UAVs), as a new way to acquire remote sensing data, has become a new technology to study earth characteristics and the properties of objects near the surface [1]. Different from traditional aircraft or satellite remote sensing platforms, UAVs can generate ultra-high-spatial-resolution digital images in a relatively low spatial range. In addition, compared with large remote sensing platforms, UAVs have faster response capability, shorter preparation time and lower operating costs [2]. UAV remote sensing is widely used in surveying and mapping, agriculture, environmental monitoring, resource surveys and military fields [3,4,5,6]. The rapid development of UAV remote sensing technology significantly reduces the cost of remote sensing data collection in a small range. A large number of UAV remote sensing public data sets that can be applied in computer vision tasks, such as target recognition, image classification and semantic segmentation, have been published one after another [7,8,9]. The convenience of UAV remote sensing provides opportunities for land cover mapping of different spatial ranges and different spatial resolutions. The use of lidar sensors can obtain vegetation height and structure information, which is useful in land cover classification [10,11].

At present, the deep learning algorithm is widely used in the field of remote sensing image classification. Zhu et al. used multi-spectral remote sensing image data from UAVs and artificial neural networks to classify vegetation on the riverbank and calculate the Simpson and Shannon–Wiener diversity index of the mountain vegetation community [12]. A Aeberli et al. combined a convolutional neural network with the local maximum filtering method to extract a single banana tree based on multi-temporal and multi-spectral remote sensing data from UAVs. The research results showed that the method had a high plant detection rate and was suitable for precision agriculture [13]. TD Camargo et al. used a lightweight deep residual convolutional neural network optimization algorithm and UAV remote sensing images to extract weeds in winter wheat planting areas, and the weed classification map drawn is rich in detail, which can provide data support for the precise spraying of pesticides [14]. L Hashemi-Beni et al. proposed a mapping method combining a full convolutional neural network (FCN) and regional growth (RG) when studying the extraction of flood areas from optical images [15]. To sum up, we can see that deep learning has good application prospects in remote sensing image classification tasks, which is due to deep learning’s hierarchical representation of data and powerful feature extraction ability. The architecture of deep neural networks is constantly evolving, from the initial multi-layer perceptron to shallow neural networks, from deep neural networks to wider and deeper neural networks and from a single convolutional neural network to a complex neural network with convolutional and cyclic integration. In addition, regularization and optimization promote improvements and the design of deep learning in different aspects.

The advent of advanced structures and modules, such as residual connections, attention mechanisms and Transformers, further improves the accuracy of classification results and the robustness of models. The Transformer is a brand new neural network architecture originally used for natural language processing, characterized by its self-attentional mechanism and efficient computing power. With the continuous improvement in Transformer architecture, it has been used for image and video processing tasks in addition to natural language processing [16], such as Vis-Transformer (ViT) [17] and the end-to-end Vision-Language Cross-modal model (VL) [18] for computer vision. At present, there are two design paradigms for image classification models based on a Transformer. One is the combination of a convolutional neural network (CNN) and Transformer. The core idea is to capture local features using limited receptive fields of the CNN and then use the Transformer to learn long-distance dependency. Finally, the model preserves the global and local features to the greatest extent. The other method is a pure Transformer architecture, which applies a standard Transformer directly to the image classification task and then trains the image classification model in a supervised learning manner. Encouraged by the Vis-Transformer’s performance, many researchers have applied the Transformer to remote sensing image classification tasks. Inspired by Vis-Transformer’s performance, a number of researchers have applied Transformers to remote sensing image classification tasks, and many experiments have shown that the performance gains generated by a Transformer are obvious.

In order to solve the problem that continuous subsampling in deep convolutional neural network leads to a small receptive field, local features can only be extracted and long-distance dependence cannot be established, this paper introduces a visual Transformer model, integrates a convolutional neural network with a Transformer and designs a semantic segmentation network, DE-UNet, with a dual encoder. The feature of the network is that two branch networks focus on global features and local features. Finally, the two types of features are fused to provide richer semantic information for the model.

## 2. Study Areas and Dataset

### 2.1. Study Area

**Figure 1:**
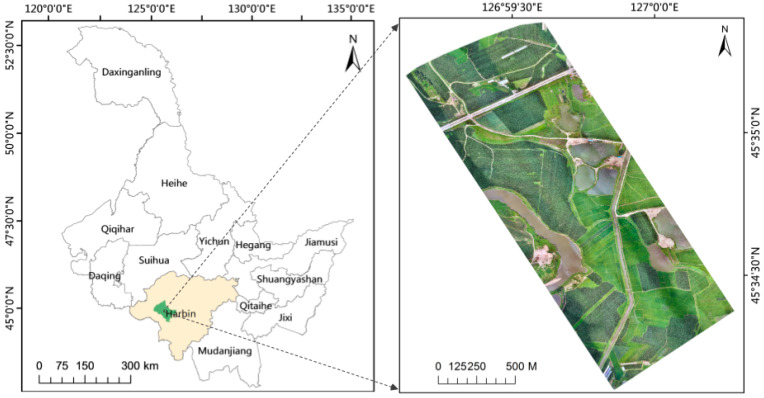
Location of the study area.

The research area is located in Acheng District, Harbin City, Heilongjiang Province, China (as shown in Figure 1). The UAV data collection range is about 1.72 square kilometers, and the altitude is between 368 m and 384 m. The main ground objects are corn, rice and water, and surrounding areas also contain shrubs and building land.

**Figure 2:**
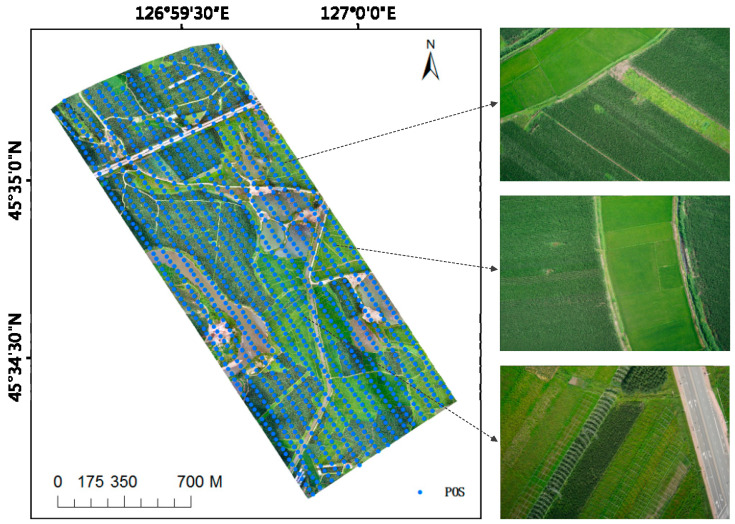
The flight path of the UAV, where each blue dot represents the location of the camera in space when the image was taken.

The multi-spectral images are taken by using a JR503 five-lens tilt photography gimlet mounted on a CW-10 composite wing UAV. The platform adopts four 40 mm tilt lenses and one 35 mm orthographic lens, with an image amplitude of 6000 × 4000 and up to 120 million pixels. It can output five channels of POS information at the same time to accurately record the exposure data of each camera. The spatial resolution is 0.04 m, the image is stored in JPEG format and the course and side overlaps are 75%. The shooting time was in the morning of 16 July 2022. The weather was clear and cloudless. The UAV flew 50 m high and took pictures with 60 m spacing and 90 m spacing. A snake-shaped route was selected, and the minimum circling radius was 120 m. The latitude of the plane is between 45°34′5.87″ N and 45°35′23.569″ N, and the longitude is between 126°59′7.569″ E–127°0′16.853″ E. The course setting and partial orthophoto data are shown in Figure 2.

### 2.2. Construct Training Sets and Testing Sets

In the indoor operation phase, we used Agisoft Metashape and Pix4Dmapper to generate orthopoeia and digital surface model (DSM) from the drone data. The steps are as follows: prior to image processing, check the point position correspondence between images and POS (coordinates corresponding to the center point of each image) information, and delete the images taken automatically during takeoff and landing without corresponding POS points, as well as the photos taken at the end of the airline and during the adjustment of UAV flight height. Finally, 1800 ortho images were obtained, and a TIF image with spatial reference was output using the ortho Mosaic module of Pix4Dmapper. The elevation system was Dalian elevation system, and the projection coordinate system was WGS_1984_UTM_Zone_52N.

**Figure 3:**
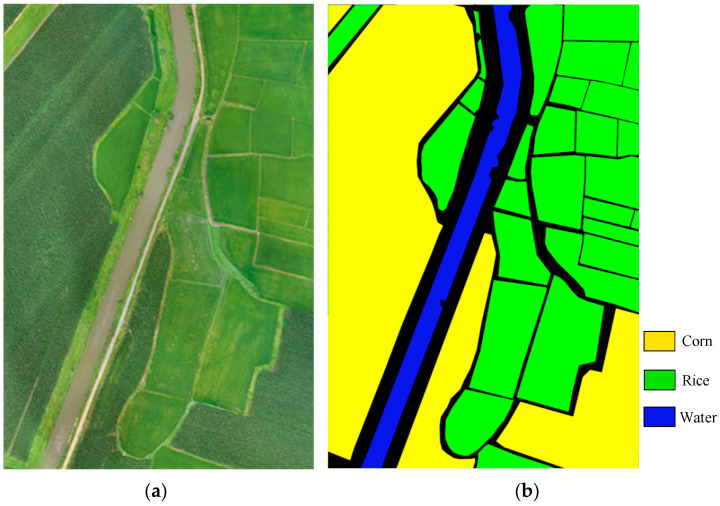
(**a**). Original UAV image (**b**). Ground truth.

Corn, rice and water accounted for more than 90% of the whole research area. Therefore, this paper constructed training sets and test sets based on these three types of ground objects. The generated UAV images have a high spatial resolution. Combined with field mapping, high-precision digitization results of ground object labels can be produced with the image as the background. This part of work is completed under ArcGIS and ENVI platforms. The training set is used for model training. First, an area of about 72,000 square meters is selected from the research area as the training set (see Figure 3). The training data are a true-color image with a spatial resolution of 12,950 × 8686, and 2000 images of 224 × 224 size are randomly cut from the original image and label data. The training samples of this semantic segmentation task are constituted. The number of training samples of various ground objects is shown in Table 1. The size of the test set is 12,548 × 8060. In order to better test the generalization ability of the deep learning model, the test set and the training set do not overlap in space. Since the dimension of the input image of the model is 224 × 224 × 3, from the upper-left corner of the test set to the lower-right corner of the test set, according to the step of 112 pixels, the size of 224 × 224 images is clipped successively. After model prediction, the predicted images are spliced to restore the initial size of the test set. The number of samples in the test set is shown in Table 2.

## 3. Methodology

In this paper, four classification algorithms are used to classify UAV image land cover, which are the traditional machine learning algorithm AdaBoost, two common semantic segmentation networks, UNet and UNet++, and our proposed DE-UNet.

### 3.1. Vision Transformer and Swin Transformer

Transformer network architecture is widely used in the field of natural language processing (NLP). Due to its excellent performance, Transformer network architecture has been introduced into the field of natural image and is currently a research hotspot in visual recognition tasks. Both Transformer and visual Transformer are based on the attention mechanism architecture, which is widely used in convolutional neural networks. The attention-enhanced convolutional network [19] connects the convolutional feature mapping with a set of feature mappings generated via self-attention to enhance the ability of the model to capture long-distance information. A large number of experiments show that the classification accuracy of the model is higher than that of the squeezing and excising network (SENet) when the number of parameters is similar, which verifies that the self-attention mechanism can enhance CNN. In the target detection task model DERT [20], only CNN is used to extract basic features, and Transformer is fully used in encoder and decoder parts, forming an encoder–decoder sequence prediction framework based on Transformer to predict all targets at one time. Vision Transformer (ViT) [16] completely gives up CNN and reuses Transformer architecture in natural language processing to solve the image problem. The input image is divided into several patches and then input into subsequent Transformer encoders in the form of a sequence. TNT (Transformer in Transformer) [21] model improves ViT by dividing patch in ViT into sub-patches and representing images with finer granularity. Research shows that TNT model has stronger learning ability and generalization ability. Swin Transformer [22] proposed hierarchical Transformer, which divides patch into windows and adopts local attention mechanism between windows. Meanwhile, in order to solve the interaction problem of information in windows between different layers, the window migration strategy is adopted to support cross-window connection between upper and lower layers. This layered design allows for great flexibility in modeling features at different scales.

**Figure 4:**
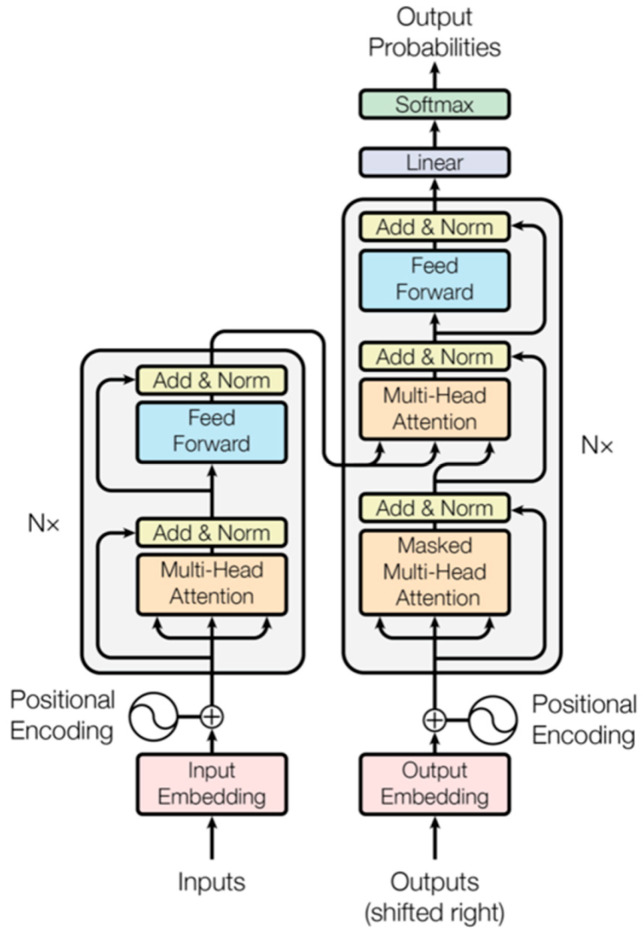
Structure of the original transformer [16].

Transformer adopts an encoder–decoder architecture and consists of three components: multi-head attention layer, feedforward neural network and layer standardization. Its structure is shown in Figure 4.

In self-attention layer, three matrices, namely query vector *Q* (Query), key vector *K* (Key) and value vector *V* (Value), are converted from the average input vector. First, *Q* and *K* are dot products and usually the calculated result is divided by $\sqrt{d_{k}}$ to prevent the result from being too large, where *d_k_* is the dimension of key vector *K*, and softmax is used to convert the result into probability distribution and then multiplied by matrix *V* to obtain the representation of weight summation, generating attention and vectors with greater probability to obtain extra attention. The calculation process is as follows:

(1) $$S=Q\cdot K^{T}$$

(2) $$S_{n}=\frac{S}{\sqrt{d_{k}}}$$

(3) $$P=Softmax\left(S_{n}\right)$$

(4) Z=V·P

The complete calculation formula can be expressed as:

(5) $$Attention\left(Q,K,V\right)=softmax\left(\frac{Q\cdot K^{T}}{\sqrt{d_{k}}}\right)\cdot V$$

In Transformer, the self-attention layer is improved by using multi-head self-attention mechanism. Single-head self-attention limits the model’s ability to focus on multiple specific spatial locations. Multi-head attention uses different query matrices, key matrices and value matrices, which are randomly initialized and trained to project input vectors into different subspaces. Thus, the model can focus on multiple positions in different subspaces and generate further attention. The calculation process can be expressed by the following formula:

(6) $$MultiHead\left(Q^{\prime },K^{\prime },V^{\prime }\right)=Concat\left(head_{1},head_{2}\ldots \ldots ,head_{h}\right)W^{O}$$

(7) $$head_{i}=Attention\left(QW_{i}^{Q},\;KW_{i}^{K},\;VW_{i}^{V}\right)$$

In the formula, the calculation process of $W_{i}^{Q}\in {\mathbb{R}}^{d_{model}\times d_{k}}$, $W_{i}^{K}$ and $W_{i}^{V}$ is similar to that of $W_{i}^{V}$, where *h* is the amount of attention. Under the multi-head attention mechanism, each attention group maintains its own input and output weight matrix. The feedforward neural network is composed of two fully connected layers. The ReLU activation function is used after the first fully connected layer. There is a feedforward neural network in the encoder and decoder but they do not share parameters.

(8) $$FN=max\left(0,X\cdot W_{1}+b_{1}\right)W_{2}+b_{2}$$

There is a residual connection between the self-attention layer of the encoder and the feedforward neural network, after which another layer standardization operation is carried out. The calculation process is as follows:

(9) $$output=LayerNorm\left(X+Attention\left(X\right)\right)$$

where *X* is the input of self-attention layer, and query vector *Q*, key vector *K* and value vector *V* are calculated from *X*.

**Figure 5:**
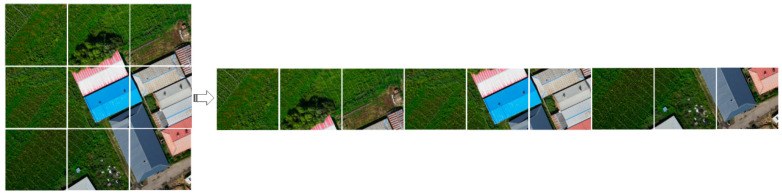
The embedding layer divides the image into equally sized parts.

The author of ViT believes that images can also be input into Transformer in the form of sequence–sequence just like word vectors in natural semantic processing, and the image patch sequence can be directly applied to Transformer to realize the classification of the whole image. The ViT model is composed of three parts: the image embedding layer, Transformer encoder and MLP. The embedding layer cuts the input image into patches of the same size. For a given image *H* × *W* × *C*, the number of images *N* is:

(10) $$N=\frac{H}{p_{1}}\times \frac{W}{p_{2}}$$

where *p*_1_ and *p*_2_ are the height and width of the image, respectively. In practical application, *p*_1_ = *p*_2_ is usually set. The input of Transformer is a two-dimensional matrix. Therefore, it is necessary to first transform the three-dimensional image into two-dimensional input of (*N*, *D*). As shown in Figure 5, the embedding layer will transform the image into sequence data that can be processed by Transformer structure. Then, each patch is mapped to a one-dimensional vector, usually called Token, through linear mapping, and each Patch corresponds to a Token. Meanwhile, according to the position of patch in the input image, we add position information identified by the vector and finally input it into the Transformer encoder. Within the embedding layer, the process of mapping patch to one-dimensional vector is realized via the convolution layer. It can be seen that ViT belongs to the design paradigm combined with CNN and Transformer.

**Figure 6:**
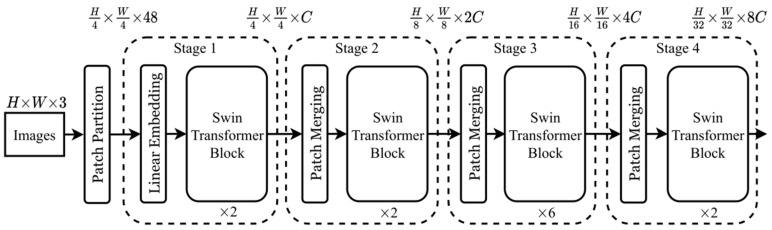
The structure of the Swin Transformer (image from [22]).

After secondary feature extraction in Transformer, the final classification results can be obtained through multi-layer perceptron, which consists of a fully connected layer and activation function. Through the above analysis, we can understand that the image resolution processed using ViT is single and fixed. Even if global self-attention has global modeling ability, it cannot obtain multi-dimensional features, and global self-attention significantly enhances the computational complexity. In order to extract multi-scale features and reduce the amount of computation, Swin Transformer model proposes hierarchical feature representation and window self-attention. The Swin Transformer architecture is shown in Figure 6.

Swin Transformer is composed of 4 stages, each of which has similar functional units. In the first stage, images are first divided into patches of the same size and then input into Swin Transformer block. In the second stage to the fourth stage, the input patch is first merged, and four adjacent patches are merged into one patch. With the deepening of the network, the size of the feature map gradually decreases, and the perception range of each patch expands, so that multi-scale features can be extracted. This operation is similar to pooling in the convolutional neural network. Window self-attention is composed of two modules: standard window self-attention and moving window self-attention. The former limits the calculation of attention to a single window, thus reducing the amount of calculation, and the latter is set to solve the information interaction between windows.

### 3.2. Semantic Segmentation Network DE-UNet

**Figure 7:**
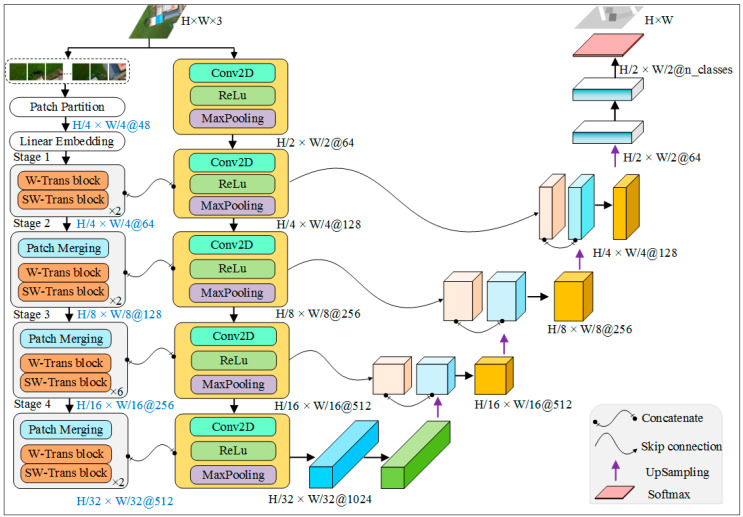
DE-UNet: a semantic segmentation network with dual encoder.

Swin Transformer, with its excellent architecture design and efficient calculation, has become a popular backbone network. It has been widely used in computer vision tasks, especially in a series of visual downstream tasks, such as image segmentation, target detection and image classification. P Kyeong-Beom et al. [22] combined Swin Transformer with CNN to propose a semantic segmentation network, SwinE Net, for medical images. Xu X et al. [23] designed the spatial attention staggered cascade network framework SAIEC based on Swin Transformer, and they proved the effectiveness and feasibility of the model in remote sensing image target detection data set. Cui Zhang et al. [24] proposed SwinSUNet, a pure Transformer model with U-shaped structure, using Swin Transformer block. Experimental results show that SwinSUNet performs better than the traditional CNN method in the task of change detection. Inspired by these models, we combine Swin Transformer and UNet to propose a DE-UNet model with dual encoder, making Transformer’s long-distance feature capture capability complementary to CNN’s local feature extraction capability. Figure 7 shows the model architecture of DE-UNet.

On the far left of DE-UNet is the Swin-Transformer-based encoder, which consists of four stages, with two Swin Transformer blocks in the first, second and fourth stages. There are six Swin Transformer blocks in the third stage. Inside each Swin Transformer block are standard windows (W-Trans block) and moving windows (SW-Trans block). The middle part is UNet network encoder, which consists of two-dimensional convolution, activation function ReLU and maximum pooling. First, in Swin Transformer encoder, the input RGB image is divided into 4 × 4 patches, and each patch has a feature dimension of 48. After reshaping each patch into a vector, a linear embedding layer is applied to record the position information of each patch. After the first stage, we output the feature map ((*H*/4 × *W*/4) × 64), that is, the height and width are one quarter of the input image, and the number of features is 64. The features of the output are passed to the next second stage and are fused with the features extracted from the second convolution unit of the UNet encoder and input to the next convolution unit. Similar operations also occur between the following stage and the convolutional unit. It needs to be noted that there is a Patch Merging module in the second to fourth stages, which downsamples input features, reduces resolution and adjusts the number of features, and its function is similar to the pooling operation in the convolutional neural network. As can be seen from Figure 7, the two encoders carried out feature fusion for a total of 4 times. With the increase in network depth, the image resolution is further reduced, and the number of features is constantly increased. After the first to fourth fusion, the output features are: ((*H*/4 × *W*/4) × 128), ((*H*/8 × *W*/8) × 256), ((*H*/16 × *W*/16) × 512) and ((*H*/32 × *W*/32) × 1024). On the right side of DE-UNet is the decoder of the model, which decodes the features extracted by the encoder. The image resolution is gradually restored to *H* × *W* through four upsampling operations (purple arrow in Figure 7). Finally, the activation function Softmax outputs the classification results. The design of dual encoder makes up for the shortcomings of convolutional neural network and makes full use of feature information of different levels, which helps to segment image more accurately. In the next part of the experiment setting, we apply the DE-UNet model to the semantic segmentation task based on the UAV remote sensing image with high spatial resolution and evaluate the performance of the model.

### 3.3. Baseline Classification Models

Two typical CNN semantic segmentation models and one machine learning model were developed for comparison.

#### 3.3.1. AdaBoost

AdaBoost (Adaptive Boosting) is an ensemble learning algorithm proposed by Freund and Schapire [25], which controls deviation and variance and has an adaptive resampling technique with enhanced prediction ability [26]. When a single linear classifier (weak classifier) cannot complete complex classification, multiple linear classifiers can be connected to form a strong classifier, which is the basic idea of AdaBoost. During operation, each sample of the training set is trained and assigned the same weight *w*, which constitutes vector *D* [27]. The weight distribution formula is shown as follows:

(11) $$D_{l}=\left(w_{l1},\;w_{l2},\;\ldots ,\;w_{lN}\right),\;w_{l1}=\frac{1}{N},\;i=1,2,\ldots ,N$$

The weak classifier *G*(*x*) is obtained by using training samples with weight distribution *D_m_* for learning:

(12) $$G_{m}\left(x\right):\;\chi \to \left\{-1,1\right\}$$

Then, in the iterative process, the sample weight is constantly adjusted to reduce the weight of samples with correct classification and increase the weight of samples with wrong classification, and the model is guided to learn again to continuously improve the prediction accuracy. AdaBoost assigns a weight value α to each weak classifier. These weights are obtained by calculating the error rate *e* of the weak classifier. The formula for calculating α is as follows:

(13) $$\alpha_{m}=\frac{1}{2}\log\frac{1-e_{m}}{e_{m}}$$

The strong classifier is based on the weak classifier. The linear combination of the weak classifier and the final strong classifier can be expressed via the following formula:

(14) $$f\left(x\right)=\overset{M}{\underset{m=1}{\sum }}\alpha_{m}G_{m}\left(x\right)$$

(15) $$G\left(x\right)=sign\left(f\left(x\right)\right)=sign\left(\overset{M}{\underset{m=1}{\sum }}\alpha_{m}G_{m}\left(x\right)\right)$$

AdaBoost has been proved to be an effective method for land cover classification [28,29,30]. In the experiment in this section, we use Python language and machine learning library scikit-learn to implement AdaBoost classifier. The base learning machine adopts decision tree. The maximum number of iterations was set to 400, and the learning rate was set to 0.8. The algorithm parameter used SAMME.R, which converges faster.

#### 3.3.2. UNet

UNet is a deep neural network proposed by Ronneberger et al. [31] for medical image segmentation. It is considered to be a classical framework in semantic segmentation tasks, which is not only applicable to binary classification but also has excellent performance in multi-classification tasks, especially when there is imbalance between categories [32]. The core of UNet architecture is downsampling, upsampling and skip connection. The shallow network is used to extract the primary features of the image, while the deep network captures the advanced features, and skip connection realizes the splicing of the two levels of features. With its simple and efficient architecture design and good adaptability, it is widely used in many computer vision tasks, such as image classification and target detection. There are also a number of variations based on the UNet architecture. For example, Ozgun Cicek et al. extended UNet architecture to design 3D UNet, which can learn from sparsely annotated stereoscopic images and test the model’s performance on complex 3D images without the use of pre-training network, achieving good segmentation results [33]. Wagner et al. used UNet to extract the forest types and tree species maps of 1600 square kilometers of tropical rain forest from the WorldView-3 satellite high-resolution image (0.3 m). The overall classification accuracy exceeded 95%, and the intersection ratio (IoU) reached 0.96 [34]. Shi et al. improved UNet and proposed CloudU-Net [35] for day–night satellite cloud image segmentation by introducing extended convolution and fully connected conditional random field (CRF). Experimental results show that the segmentation effect of this network is better than UNet and FCN. Li et al. proposed MACU-Net using asymmetric convolution and multi-scale skip join, which achieved ideal classification accuracy in high-resolution remote sensing image segmentation [36].

#### 3.3.3. UNet++

**Figure 8:**
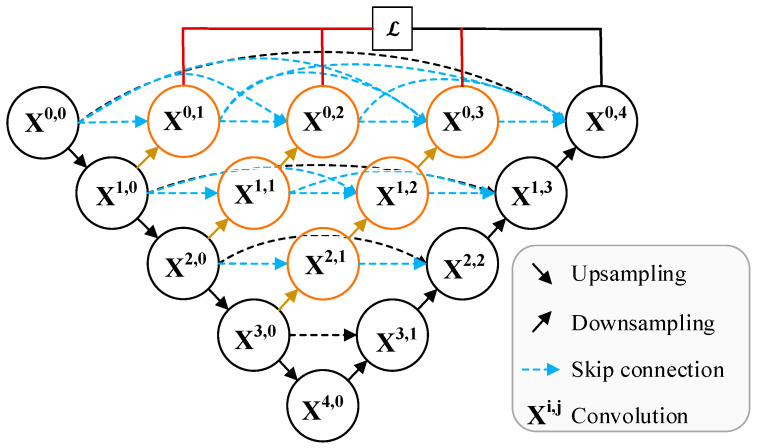
The architecture of UNet++.

UNet++ [37] improved the UNet architecture by using more dense skip connections, so as to reduce the semantic loss of the feature map in the process of encoding and decoding and make full use of the multi-scale features of the image. The architecture of UNet++ is shown in Figure 8. The yellow and blue arrows represent the intensive connections added by the model. Meanwhile, in order to obtain more adequate training for the shallow network and solve the problems, such as the disappearance of training gradient and slow convergence rate, deep supervision (red line) is also added to the model.

Similar to UNet, there are also many variations of UNet++, and it is widely used in high-resolution remote sensing image classification and target detection tasks. Some experiments show that UNet++ with deep supervision mechanism has obvious performance gain compared with UNet [38,39,40].

## 4. Experimental Results and Discussion

### 4.1. Model Training

We used Python 3.7 and the deep learning framework Keras to construct the DE-UNet network. DE-UNet was compared with AdaBoost and another two deep learning models (UNet and UNet++). In order to fairly compare the impact of different methods on the classification results, the three deep learning models used the same hyperparameter settings during training.

**Figure 9:**
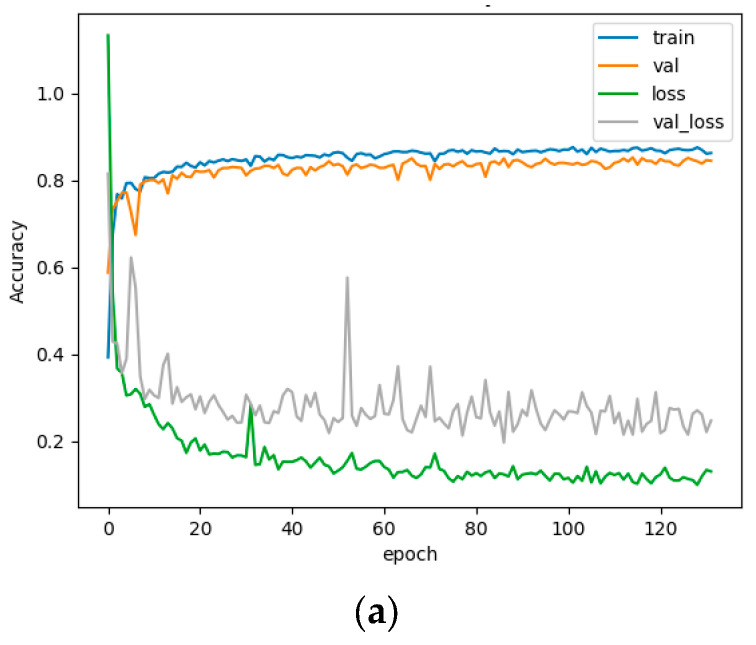

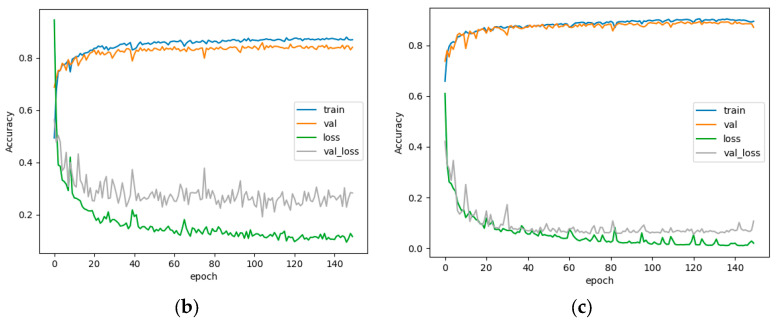
Loss and precision change curves of three deep learning algorithms during training. (**a**) UNet; (**b**) UNet++; (**c**) DE-UNet.

The learning rate is 0.05, the batch size is set to 16, the weight attenuation is 0.001, the epoch is 150, Adam is used as the optimization function and the early stopping strategy is used in the entire training process. Training is stopped when the training accuracy does not increase for 30 consecutive rounds. The training process when the deep learning model achieves the highest classification accuracy is shown in Figure 9. As can be seen from Figure 9, the three deep learning models all converge to a higher accuracy but the convergence speed is slow. Among them, UNet has an early stop phenomenon, and no fitting occurred in all models.

### 4.2. Classification Result and Evaluation

In order to better compare the performance of different classification algorithms, we trained each algorithm five times in the experiment and then calculated the average classification accuracy and standard deviation of various ground objects on the test set. Table 3 summarizes the average evaluation indexes obtained via different classification algorithms in five runs.

**Figure 10:**
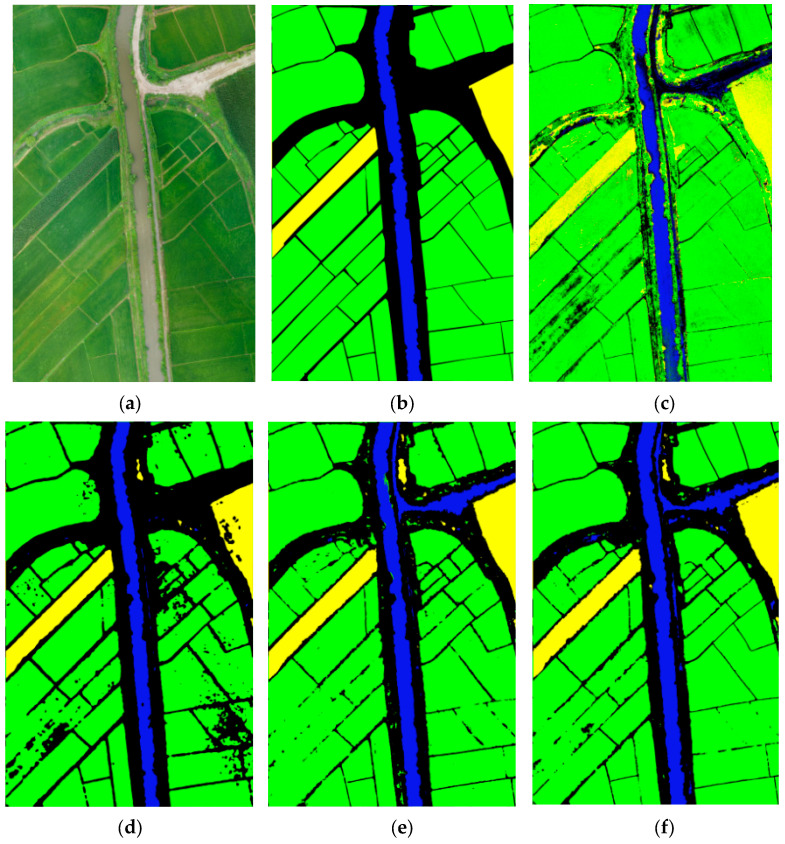
Classification results of 4 different classification algorithms (**a**). Original UAV image (**b**). Ground truth (**c**). AdaBoost (**d**). UNet (**e**). UNet++ (**f**). DE-UNet.

The statistical results in Table 3 show that the proposed DE-UNet model has the best performance, and the classification accuracy of the two crops and the overall classification accuracy are better than the other three algorithms. In five training sessions, the highest classification accuracy obtained using DE-UNet on corn and rice is 98.47% and 98.24%, respectively. The performance of UNet++ is slightly better than that of UNet. In terms of the classification accuracy of water bodies, UNet++ performs better but is not stable enough. The overall classification accuracy of the traditional machine learning algorithm, AdaBoost, is relatively low, especially in the recognition of corn and water bodies. The average classification accuracy of corn is only about 50% for five times. For different methods achieving the highest overall classification accuracy, the corresponding classification map is shown in Figure 10.

It can be seen from the classification map that there is a lot of noise in the classification results of AdaBoost, and the algorithm misclassification weeds on both sides of the river to rice and bushes on both sides of the road to corn. The classification effect of the three deep learning models on corn and rice is ideal, and the classification accuracy is over 90%. However, in the classification of water bodies, only UNet++ has a good performance. When marking ground objects, we take roads and bushes as the background, which poses a challenge to the model because there is a small amount of water on the road surface in this region. UNet++ and DE-UNet divided some waterlogged roads into water bodies, which affected the average classification accuracy and the overall classification accuracy.

By comparing the classification results of maize and rice, we found that the automatic extraction results of the two crops are highly consistent with the actual distribution, and the land boundaries are clear. However, there is obvious misclassification between water bodies and waterlogging roads and between water bodies and buildings, which is related to the fact that we did not label the roads and buildings in categories, so the model cannot effectively learn the features of these two types of ground objects.

After years of development, the control of a UAV aerial photography platform is becoming more and more simple, the data acquisition method is more flexible and efficient, the data quality is constantly improved and the cost of data acquisition is much lower than that of traditional aerial photography technology. Classification results based on UAV remote sensing images can be widely used in natural disaster assessments, planting area estimations and other applications.

### 4.3. Discussion

In this work, we proved that UAV images are suitable for land cover classification. This paper compared the traditional machine learning classification algorithm with the current popular advanced deep learning algorithm. The experimental results show that the traditional machine learning algorithm is not always good at dealing with the unbalanced classification problem. The deep learning classification method based on nonlinear input transformation (UNet, UNet++, DE-UNet) has better performance than the machine learning method (AdaBoost).

Among the three deep learning methods, DE-UNet with the dual encoder, proposed in this paper, is better than UNet and UNet++. As can be seen from Table 3 and Figure 10, the classification accuracy of both corn and rice is very high for the deep learning model, and the high misclassification rate between water body and background is the main issue affecting the overall classification accuracy of the model. In addition, as expected, the addition of a Swin Transformer significantly improves the classification accuracy for corn and rice. Although the deep learning method has obvious performance advantages in land cover classification using UAV remote sensing, there are still two limiting factors in the practical operation. First, the imbalance of samples resulted in low training accuracy and prediction accuracy of the model, which was specifically reflected in the obvious difference between the classification accuracy of the water body and the two crops. Second, there are many parameters in the deep learning model, so training a deep learning model requires a large computational cost. In the experiment, GPU (8G) acceleration is used in this study, and the training time of UNet, UNet++ and DE-UNet is about 5, 11 and 16 h, respectively, while the training time of AdaBoost is 2 h. How to balance the efficiency and precision of the model is one of the research directions in our follow-up work.

In this study, in addition to the proposed DE-UNet, we also chose UNet and UNet++, two commonly used deep learning supervised classification algorithms, which are widely used in the field of remote sensing image classification and also achieved satisfactory accuracy in this classification task. All these indicate that in the field of remote sensing image classification, the technology of intelligent extraction of image features using convolutional neural networks or vision Transformers is very important. The combination of the two is a widely used architecture design nowadays. It can even be predicted that a new computer vision application paradigm based on a vision Transformer will be formed in the future.

## 5. Conclusions

Deep learning has been widely used in the field of remote sensing. This paper studies the application potential of UAV remote sensing data in land cover maps and evaluates the latest deep learning supervised classification algorithm. We propose a semantic segmentation network that integrates a convolutional neural network and Swin Transformer, and we evaluate its performance on training sets and test sets. The experimental results show that compared with traditional machine learning methods, the three deep learning methods have consistent improvements in classification accuracy, the proposed DE-UNet method performs the best and the hybrid architecture of the Swin Tranformer fusion convolutional neural network has more powerful feature extraction ability. It was observed that, using the DE-UNet method, the average classification accuracy reached 96.07% for corn and 98.46% for rice. Meanwhile, the other two deep learning methods also achieved high classification accuracy.

It is worth mentioning that the experiment was conducted in July and the data were single-phase UAV images with high vegetation coverage. However, vegetation at different growth stages is different in spectral curves, spatial textures and other features, which challenges the generalization ability of the model. When the growth stage of vegetation changes, the model may not be able to give satisfactory results. A common method is to solve this problem through multi-temporal remote sensing, which is what we will pay attention to in future work.

Due to cloud cover, shadow generated by tall ground objects and random noise generated in the process of image acquisition, the quality of UAV images will be affected, which poses a great challenge to the classification algorithm, thus affecting the classification results. For oblique photogrammetry, in addition to obtaining visible light images, point cloud data of ground objects can also be generated. The data fusion of point cloud and visible images to generate new 3D scenes and classification based on a deep learning algorithm is the main direction of our future research.

## Figures and Tables

**Table 1:** Training samples.

No.	Class	Color	Sample Size
1	Corn	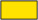	46,083,620
2	Rice	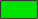	41,024,595
3	Water	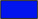	6,814,415

**Table 2:** Test samples, where the resolution of the test set was 12,548 × 8060.

No.	Class	Color	Sample Size
1	Corn	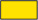	5,874,169
2	Rice	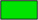	64,240,779
3	Water	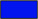	5,591,738

**Table 3:** Classification accuracy obtained by 4 different classification algorithms.

Class	AdaBoost Mean ± SD	UNet Mean ± SD	UNet++ Mean ± SD	DE-UNet Mean ± SD
Corn	48.69 ± 0.07%	94.32 ± 1.82%	96.02 ± 3.53%	96.07 ± 2.08%
Rice	85.83 ± 0.11%	96.92 ± 0.60%	98.36 ± 0.67%	98.46 ± 0.54%
Water	76.18 ± 0.24%	76.89 ± 3.10%	93.23 ± 6.56%	74.08 ± 1.17%
OA (%)	71.12 ± 0.05	94.23 ± 0.48	89.70 ± 1.80	94.51 ± 0.94
AA (%)	79.53 ± 0.03	90.20 ± 0.86	90.53 ± 1.86	88.74 ± 3.03
Kappa × 100	67.60 ± 0.02	89.08 ± 0.92	81.13 ± 3.60	89.74 ± 1.66

## Data Availability

All data sets used and produced for the purposes of this study can be requested from the corresponding author.
